# MiR-21 Expression in the Tumor Stroma of Oral Squamous Cell Carcinoma: An Independent Biomarker of Disease Free Survival

**DOI:** 10.1371/journal.pone.0095193

**Published:** 2014-04-22

**Authors:** Nora Hedbäck, David H. Jensen, Lena Specht, Anne-Marie K. Fiehn, Marianne H. Therkildsen, Lennart Friis-Hansen, Erik Dabelsteen, Christian von Buchwald

**Affiliations:** 1 Department of Otorhinolaryngology, Head and Neck Surgery and Audiology, Rigshospitalet, University of Copenhagen, Copenhagen, Denmark; 2 Department of Oncology, Rigshospitalet, University of Copenhagen, Copenhagen, Denmark; 3 Department of Pathology, Rigshospitalet, University of Copenhagen, Copenhagen, Denmark; 4 Centre for Genomic Medicine, Rigshospitalet, University of Copenhagen, Copenhagen, Denmark; 5 Department of Oral Health Sciences, Faculty of Health Sciences, Panum Institute, University of Copenhagen, Copenhagen, Denmark; Deutsches Krebsforschungszentrum, Germany

## Abstract

Oral squamous cell carcinoma (OSCC) patients have a high mortality rate; thus, new clinical biomarkers and therapeutic options are needed. MicroRNAs (miRNAs) are short noncoding RNAs that regulate posttranscriptional gene expression and are commonly deregulated in OSCC and other cancers. MicroRNA-21 (miR-21) is the most consistently overexpressed miRNA in several types of cancer, and it might be a useful clinical biomarker and therapeutic target. To better understand the role of miR-21 in OSCC, paraffin-embedded tumor tissue samples from 86 patients with primary OSCC were analyzed by in situ hybridization. We found that miR-21 was primarily expressed in the tumor stroma and in some tumor-associated blood vessels with no expression in the adjacent normal epithelia or stroma. Using image analysis, we quantitatively estimated miR-21 expression levels specifically in the stroma of a cohort of OSCC samples. These miR-21 levels significantly correlated with disease free survival with the highest levels being located in the stroma. Stromal miR-21 expression was independently associated with a poorer prognosis, even after adjusting for clinical parameters (perineural invasion and N-stage) in a multivariate analysis. In summary, we have shown that miR-21 is located in the carcinoma cells, stroma and blood vessels of tumors, and its expression specifically in the stromal compartment has a negative prognostic value in OSCC.

## Introduction

Oral squamous cell carcinoma (OSCC) is a serious disease with a five year overall survival of 60% [1]. Of course certain clinical and histological features are well-known for being related to a poor survival, but even when such changes are seen there are no way of identifying the clinical course with certainty. This lack of predictive features also applies to the risks associated with tumor relapse.

MicroRNAs (miRNAs) are short, single stranded, non-coding RNAs involved in the posttranscriptional regulation of target messenger RNAs. One miRNA can target multiple different mRNAs, and their expression pattern may change in different disease states. miR-21 regulates cell proliferation, apoptosis and the epithelial to mesenchymal transition during neoplastic progression and is up regulated in a variety of cancers [2]–[6]. mRNA targets of miR-21 that have been validated in cell lines in vitro include the tumor suppressors PDCD4, PTEM, TPM1 and SPRY2 [7]–[10]. Clinically, increased expression of miR-21 is associated with a poorer prognosis in tongue squamous cell carcinomas and other tumors [2]–[6], [11]–[13]. miR-21 has also been suggested as a marker for distinguishing progressive and non-progressive leukoplakia and OSCC [14]. The histological localization of miR-21 in tumors is controversial; in esophageal carcinomas miR-21 is expressed in the tumor cells [12], [13] wheras in breast and colon carcinomas miR-21 is expressed in stromal fibroblasts [5], [15], [16].

Different cancer-associated fibroblasts have been described, but the one that is most consistently shown to have an adverse effect on prognosis are myofibroblasts [17]–[19]. Myofibroblasts express α-smooth muscle actin and are frequently found in the stroma of oral carcinomas with poor prognosis and in oral wounds [17]–[21]. Based on the findings described above, it is tempting to suggest that the increased expression of miR-21 that we [22] and others [11] have demonstrated in tissue from oral carcinomas is found in myofibroblasts and not in the carcinoma cells [23].

In this study, we demonstrate that OSCC miR-21 expression is predominately localized to tumor stromal cells with colocalization between miR-21 and α-smooth muscle actin. We further demonstrate that the levels of miR-21 significantly correlate with disease free survival.

## Methods

### Patients

The Scientific Ethical Committee of the Capitol Region of Denmark, and the Data Protection Authority approved this study (ID nr: H-2-2012-050). Since a large group of patients had died or were severely ill, it was judged by the ethical committee that it would cause more distress than good to be informed about the project; it was therefore judged not to be necessary to obtain informed consent. Patient records were anonymized and de-identified prior to analysis. We selected archived tissue from 111 patients diagnosed with either tongue cancer or floor of the mouth cancer from 2000–2008 who were treated at Rigshospitalet Copenhagen, Denmark using the DAHANCA database [24]. The total cohort in this period comprises 216 patients with tongue or floor of the mouth cancer, of which the 111 patients were randomly chosen. Clinical data on all patients stem from the DAHANCA database and clinical hospital records (Table 1). The tumor samples included 21 tumors of the tongue and 65 from floor of the mouth cancer. Patients were divided into either a clear margin (histologically ≥5 mm free resection margin) or positive surgical margin group according to the pathology report [24]. Pathological review of the samples revealed that 10 of the selected cases had been misclassified as OSCCs and were excluded. In addition, sufficient tumor material could not be retrieved from 9 patient samples, and they were also excluded. Six samples were of poor technical quality and were therefore excluded. Thus, the study cohort consisted of 86 patients (described in Table 1). All 86 patients were included in the analyses mentioned below. These 86 patients did not differ significantly from the total (the original 216) with regard to age, gender or stage by a chi-square test (data not shown). We used the WHO malignancy grading system of oral cancer to divide patients into either poorly differentiated or well and moderately differentiated carcinomas [25]. Patients were treated in accordance with DAHANCA-guidelines; 68 of the patients were surgically treated and 18 received radiotherapy in addition to surgery [24].

**Table 1:** Univariate analysis of factors associated with disease free survival in OSCC.

	n	Unajusted hazard ratio (95% CI)	p-value
**Age**	86	1,04 (0.995, 1.09)	0.082
**Gender**	63	1	
	23	0.88 (0.39, 1.99)	0.77
**Site**	21	1	
	65	1.04 (0.44, 2.4)	0.92
**Relapse site**	15	1	
	10	1.64 (0.66, 4.1)	0.29
	1	1.05 (0.22, 4.8)	0.95
	2	1.56 (0.57, 13.0)	0.21
**Surgical margin**	47	1	
	39	1.88 (0.90, 3.8)	0.094
**T status**	80	1	
	6	1.29 (0.31, 5.5)	0.73
**N status**	65	1	
	21	3.0 (1.5, 6.3)	0.003*
**Grade**	75	1	
	11	1.2 (0.42, 3.5)	0.72
**Perineural invasion**	71	1	
	15	3.1 (1.5, 6.8)	0.003*
**Stage**	55	1	
	3	0	0.98
	28	2.8 (1.4, 5.8)	0.005*
**Smoking**		1	
	86	0.995 (0.98, 1.009)	0.49
**MiR-21 in tumor**	27	1	
	29	1.0 (0.41, 2.5)	0.996
	30	1.0 (0.42, 2.6)	0.93
**Area of stroma**		1	
		0.92 (0.12, 7.9)	0.94
		2.4 (0.31, 18.8)	0.41
**Mir-21 in stroma**	29	1	
	28	1.8 (0.68, 4.7)	0.25
	29	2.7 (1.1, 6.9)	0.032*

Footnote: * significant factors associated with survival.

### Tissue Preparation, in situ Hybridization and Immunohistochemistry

Formalin-fixed paraffin-embedded (FFPE) tissue was cut into 5 µM sections under RNAse free conditions and mounted on Super Frost slides. For the detection of miR-21, the miRCURY LNA microRNA ISH optimization kit (FFPE) (Exiqon, Vedbaek, Denmark) was used according to the manufacturer’s recommendations. Briefly, slides were deparaffinized and rehydrated before being placed in a hybridizer (Dako hybridization, Glostrup, Denmark). Tissue sections were treated with 15 mg/µl proteinase-K at 37°C for 8 minutes and hybridized with a digoxigenin (DIG)-labeled LNA probe at 55°C for 120 min. Slides were subsequently washed in decreasing concentrations of a saline-sodium citrate (SSC)-buffer at the hybridization temperature. Subsequently, DIG blocking reagent was applied for 15 min followed by incubation with an alkaline phosphatase-conjugated anti-DIG antibody. The probes were visualized with alkaline phosphatase substrate (NBT/BCIP tablets, Roche Diagnostics A/S, Hvidovre, Denmark) over night at room temperature. Slides were counterstained with nuclear fast red (Sigma-Aldrich, Denmark) for 1 minute at room temperature, dehydrated in alcohol and mounted with Eukitt mounting medium (Sigma-Aldrich, Denmark). The double DIG-labeled miRCURY LNA miRNA detection probes used to detect miR-21 in this study were full-length 22-mers (Exiqon, Vedbaek, Denmark).

**Figure 1:**
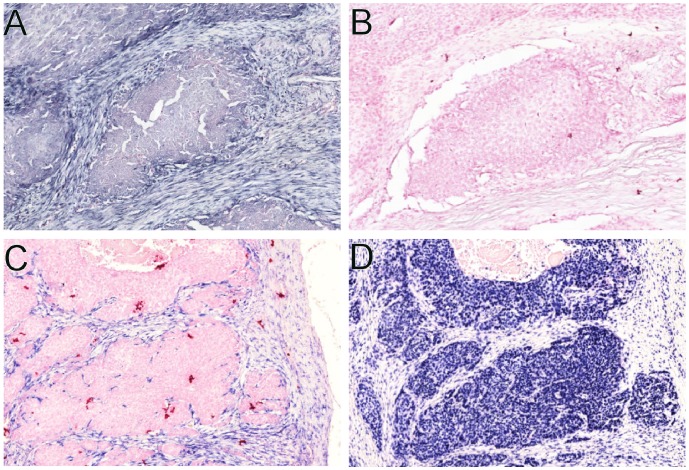
The miR-21 in situ hybridization is specific. Comparison between the miR-21 probe (A) and the scrambled probe (B) in the same tumor. The scrambled probe (B) gave no expression. Comparison between miR-21 probe (C) and U6 probe (D) in the same tumor. The U6 probe gives a positive nuclear expression in virtually every cell.

We validated the specificity of the miR-21 expression by comparing the miR-21 expression in the samples to the expression of a nonspecific sequence (scrambled), which gave no expression (Fig. 1B), thus excluding non-specific binding of the anti-DIG antibody. As a positive control, we used a probe for snRNA U6, which was present in the nuclei of nearly all cells in all samples (Fig. 1D).

Immunohistochemistry for cytokeratin was carried out using the Ventana Benchmark Ultra autostainer (Roche A/S, Hvidovre, Denmark) with the optiView detection kit and the pan-cytokeratin antibody CK-WSS (Dako, Glostrup, Denmark) in a 1:4000 dilution. The immunohistochemistry and in situ hybridization was performed on serial sections with a maximum of 5µm apart.

### Combined Fluorescent in situ Hybridization for miR-21 and Immunofluorescence for α-SMA

A total of 10 samples were randomly chosen to undergo combined fluorescent in situ hybridization (FISH) and immunofluorescence (IF) staining. The protocol for combined FISH and IF was identical to the above-mentioned ISH with a few modifications [26]. We used anti-DIG-POD instead of the alkaline phosphatase-anti-DIG probe for 60 minutes at room temperature. Slides were subsequently washed and then incubated with tetramethylrhodamine twice for 5 min at room temperature (TSA Plus TMR System, PerkinElmer, Skovlunde, Denmark). Slides were then stained for 1 hour with a α-smooth muscle actin (α-SMA) antibody followed by a secondary goat-anti-mouse Alexa Fluor 488 conjugated antibody H+L (Life Technologies Europe BV, Naerum, Denmark) (Dako, Glostrup, Denmark) for 1 hour at room temperature. The antibodies to α-smooth muscle actin and the staining procedure including controls have been described previously [21].

### Image Acquisition and Analysis

**Figure 2:**
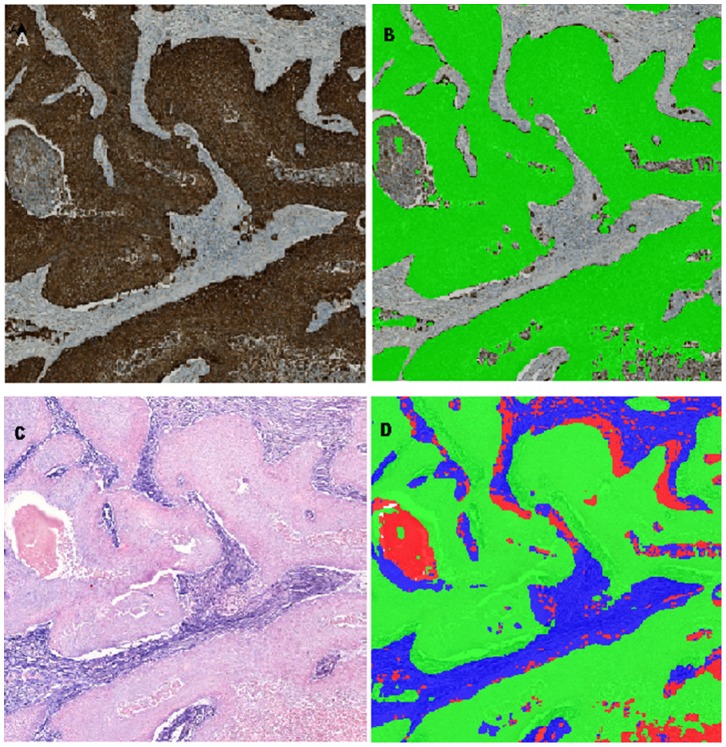
Digital image analysis of in situ hybridization. The miR-21 expression in the stroma was estimated using digital image analysis. Image (A), (B), (C) and (D) overview the image-analysis process, where (A) is the original cytokeratin stained image, which is used to delineate epithelial tumor cells, seen as green color in (B). Original image of miR-21 ISH on serial section from same tumor, where the ISH signal is quantitated specifically in the stroma area (blue signal). The red color in (B) represents stroma area negative for miR-21. See methods for a detailed description of the image analysis process.

Samples used for miR-21 in situ hybridization were scanned using the panoramic axioscan midi, (3D Histech, Histolab Products AB, Goteborg, Sweden). Samples used for cytokeratin immunohistochemistry were scanned using the Axio Scan.Z1 (Carl Zeiss A/S, Birkeroed, Denmark). The digital files were opened in the image analysis module Visiomorph in the Visiopharm software (Visiopharm A/S, Hoersholm, Denmark). The ISH and cytokeratin-stained sections were subsequently aligned using the image analysis module TissueAlign. TissueAlign is able to perform a cell-to-cell alignment of serial sections with any kind of staining, which enables so-called virtual double staining. We then manually delineated the entire tumor area on each patient sample as a region of interest, in which the subsequent analyses were performed. We used the cytokeratin stained sections to delineate tumor cells. We did this by setting a threshold for the brown, 3,3'-Diaminobenzidine (DAB), signal, to a level in which stroma was separated from the tumor, as indicated in Fig. 2A and Fig. 2B. We then subsequently set a threshold for the blue signal on the ISH stained samples, and delineated the area of miR-21 occupied only in the stromal compartment (Fig. 2C and 2D). The remaining stroma area negative for miR-21 is seen as a red color in Fig. 2D. We could then calculate the mean intensity of blue in the entire fraction of stroma positive for miR-21. To get a robust measure of the amount of miR-21, we multiplied the total area of miR-21 in the entire tumor stroma (blue area in the classified image) to the mean intensity of the blue signal, which we termed total blue signal in stroma (TBS). We subsequently performed the same analysis in the tumor cell compartment, and calculated a measure for the amount of miR-21 in the tumor cell compartment, which we termed total blue signal in tumor (TBT). Finally, we also calculated the total stromal area in our tumors, by adding the area of blue to the area of red, which represents the total area of the tumor not occupied by epithelial tumor cells, which we call total stroma area (TSA).

### Statistical Analysis

Disease free survival was defined as time from operation to recurrence. Survival curves were plotted using the Cox regression survival curves, which show relationships between miR-21 expression and disease free survival (DSF). Multivariate Cox proportional hazard regression analysis was performed using the four prognostic factors that were found to be statistically significant in the univariate analysis followed by backward elimination. The proportional hazards assumption was tested using partial residuals from the variables plotted against time to recurrence. TBS, TBT, perineural invasion, stage and N-stage fulfilled the proportional hazards assumption. Statistical analysis was carried out using SPSS 20 with an overall significance level of P<0.05. P-values from the Cox regression analysis were from log-rank tests. Correlation analyses were performed using Spearmans rank correlation.

## Results

### miR-21 is Expressed in Myofibroblasts and Other Stromal Cells in OSCC

**Figure 3:**
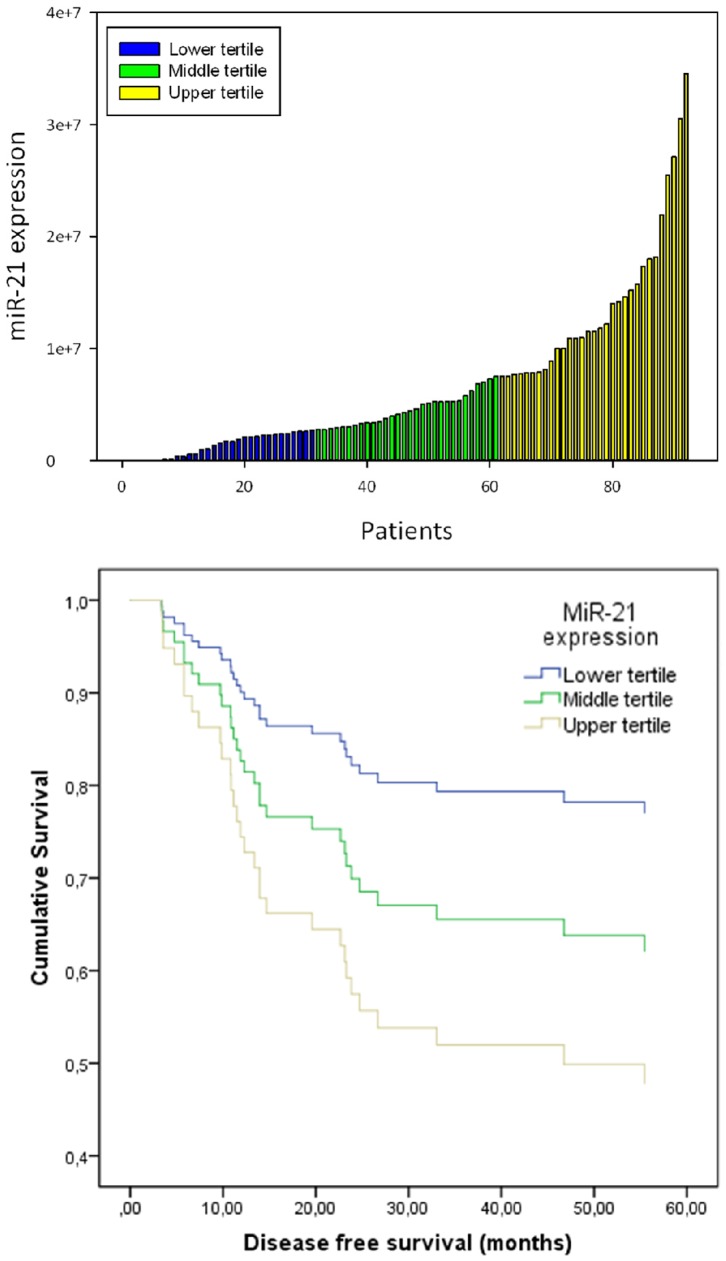
High miR-21 expression is associated with poor survival. Top: Waterfall plot of miR-21 expression in 86 OSCCs. miR-21 expression was assessed by in situ hybridization and converted to the area occupied by miR-21 (blue ) times the mean intensity of the blue signal. The cut-off between the upper, middle and lower tertile are depicted by blue, yellow and green bars, respectively. Bottom: Cox regression survival curve for disease free survival, based on miR-21 expression tertiles. Low miR-21 expression is associated with a better survival (p = 0.032).

**Figure 4:**
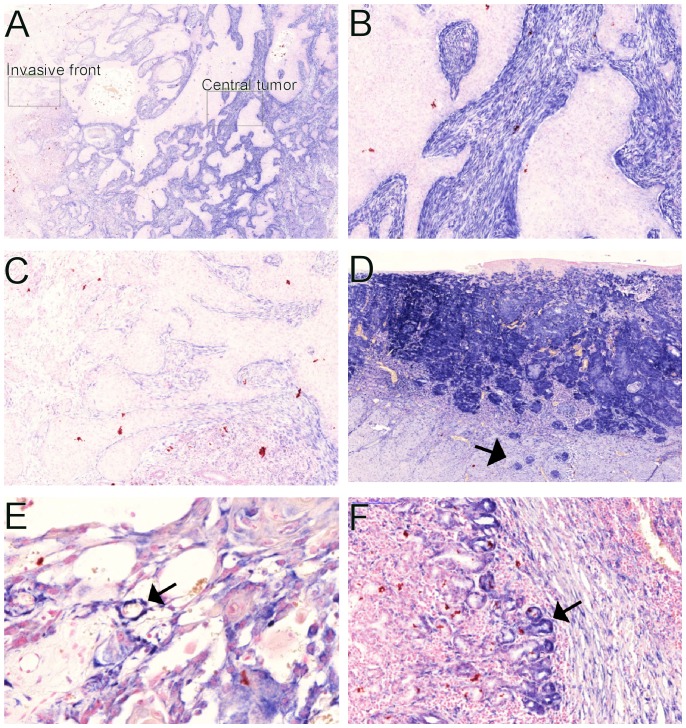
miR-21 expression is found in the tumor, endothelium and acinar cells in a subset of OSCCs. (A) An overview image showing the distribution of the miR-21 expression, which is highest in the central portion of the tumor (B) and is lower at the invasive tumor front (C). We found a strong miR-21 expression in the tumor cells from one specimen (D), which was also present in the budding tumor cells (arrow). miR-21 expression was also observed in the endothelial cells (E, arrow) and in the degenerated acinar cells (F, arrow).

We found little to no miR-21 stroma expression in 10 of the 86 samples as observed from the TBS results in Fig. 3 (top). The remaining 76 samples expressed miR-21 with varying degrees of intensity (Fig. 1A and 1C). Furthermore, 10 samples expressed miR-21 in both the tumor stroma and tumor cells, but only one sample showed an almost exclusive tumor expression (Fig. 4D) measured also as a higher TBT than TBS.

**Figure 5:**
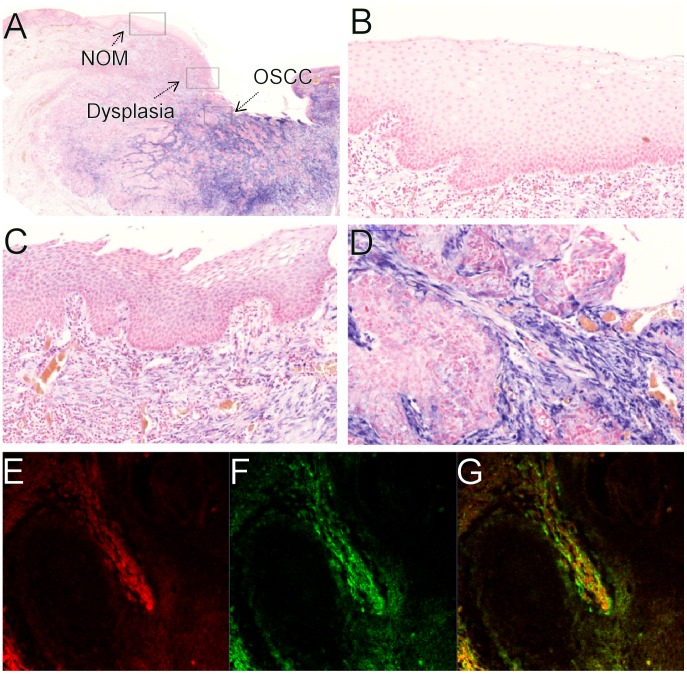
miR-21 is mainly expressed in the tumor stroma in OSCC. (A) An overview image of miR-21 expression where the areas marked by rectangles are magnified in, (B) normal oral mucosa, (C) mild epithelial dysplasia and (D) oral squamous cell carcinoma. Mir-21 appears as a blue stain. In normal oral mucosa, (B) no miR-21 expression is observed, whereas in dysplasia, (C) a weak expression is seen in the subepithelial connective tissue, and a widespread and intense expression is found in the tumor stromal compartment (D). Panels (E), (F) and (G) illustrate combined FISH and IF for miR-21 (E), α-SMA (F) and a composite image (G) showing a high degree of overlap between the two markers.

The miR-21 expression was observed in stromal fibroblast like cells (Fig. 5D) and endothelial cells (Fig. 4E). In addition, we observed miR-21 expression in salivary gland acinar cells with degeneration (Fig. 4F). The expression intensity in the carcinoma cells with one exception was markedly weaker than stromal expression (Fig. 4D). We did not observe miR-21 expression in either the stroma or the epithelial compartment from the adjacent normal oral mucosa (Fig. 5B). The stromal expression from most of the samples was abundant and occupied almost the entire tumor center (Fig. 5D and 4B). The expression of stromal miR-21 at the invasive front, which is defined as the band of tissue between the tumor front and adjacent normal tissue, was less than in the central part of the tumor (Fig. 4C and 4B, respectively), and the stroma surrounding tumor islands representing the invasive front of the tumor was often negative (Fig. 4C).

There was a high degree of overlap between miR-21 and α-SMA staining (Fig. 5G). However, we observed miR-21 positive stromal cells that were negative for α-SMA although we have not further characterized these cells. Thus, miR-21 is mainly, but not exclusively, expressed by the myofibroblast cell population of cancer-associated fibroblasts (CAFs) in OSCC.

### Association between miR-21expression in the Stroma and Clinical and Pathological Characteristics

A significant correlation between miR-21 expression in the stroma (TBS) and gender was observed, in that males had significantly higher TBS values. In addition a correlation between high TBS and an increased risk of relapse was found (Table 2). Also a borderline significant correlation between increasing differentiation grade and TBS was observed (P = 0.078). Other significant correlations between TBS and clinical characteristics were not found.

**Table 2:** Spearman correlation between miR-21 expression in the stroma (measured as TBS) and clinical variables.

Variable	n	Correlation Coefficient	p-value
**Age (yrs.)**	86	0.048	0.65
**Gender**	86	−0.27	0.009*
**Site**	86	−0.16	0.13
**Relapse**	86	0.22	0.040*
**Relapse site**	86	0.12	0.53
**Surgical margin**	86	−0.023	0.83
**T-status**	86	0.12	0.24
**N-status**	86	0.036	0.74
**Grade**	86	0.19	0.078
**Perineural invasion**	86	0.065	0.54
**Stage**	86	0.10	0.33
**Pack years**	86	−0.081	0.45

Legend: *, significant correlation. A significant correlation was found with relapse and gender.

*High stromal miR-21 expression is associated with a decreased disease free survival *We did not find any significant differences in tumor subsite with regard to clinical or pathological characteristics, and we therefore chose not to stratify survival analyses of clinical and pathological characteristics based on subsite. First, we examined the relationship of miR-21 expression and disease free survival and chose to divide patients into tertiles based on their TBS (Fig. 3, top). The relationship between disease-free survival and miR-21 expression was analyzed using Cox regression survival curves (Fig. 3, bottom). The survival curve shows that patients in the highest tertile of miR-21 expression had a significantly shorter disease free survival when compared to the patient group in the lowest tertile of miR-21 expression (P = 0.032).

**Figure 6:**
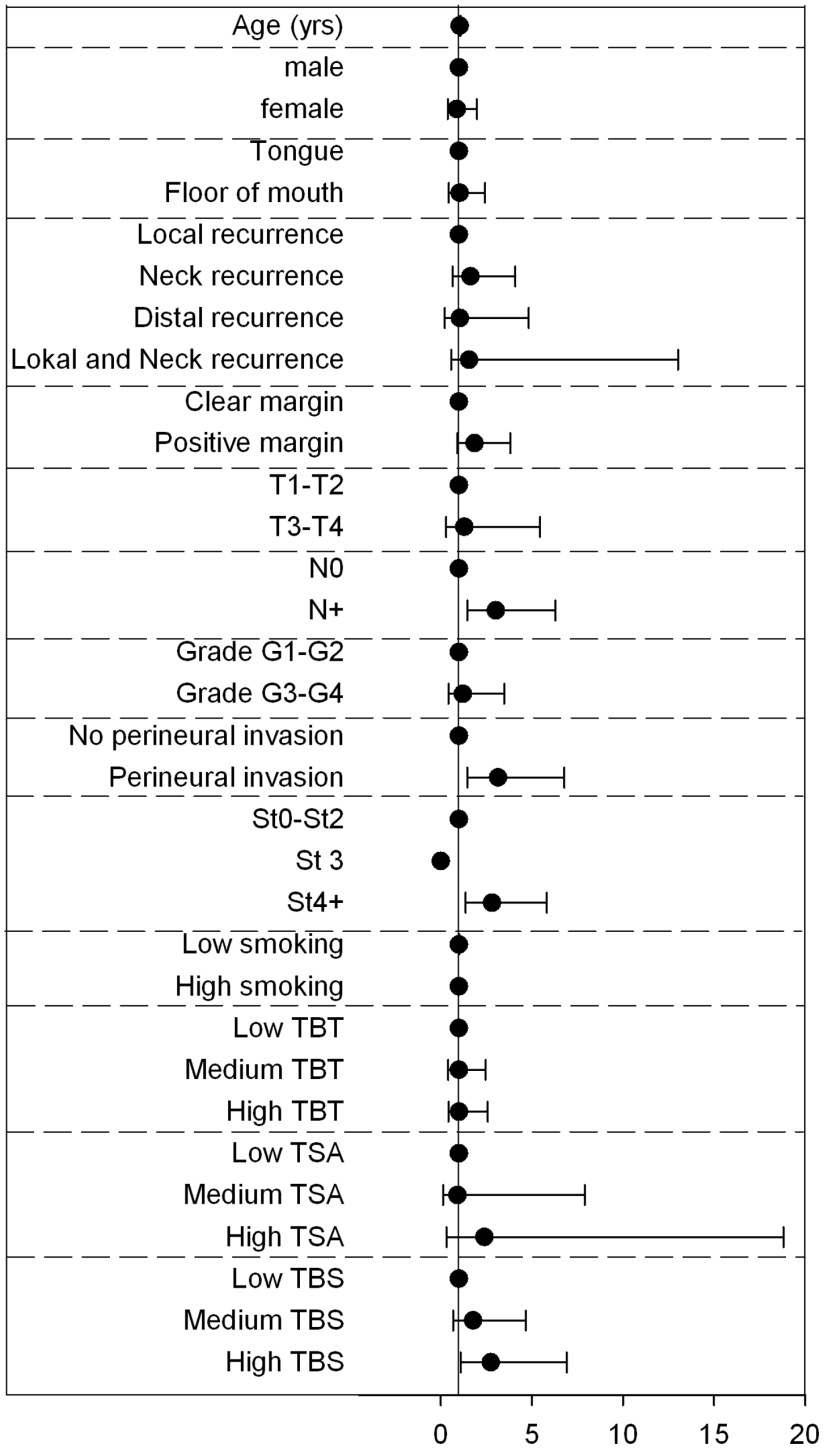
Hazard ratios from univariate analysis for disease free survival in OSCC. On the right hazard ratios including 95% confidence intervals are displayed. See Table 1 for details.

We also examined the hazard ratio for disease free survival (DFS) in relation to clinical and histological characteristics (Table 1 and Fig. 6). Only nodal spread, perineural invasion and high stage were associated with a reduced disease free survival. Gender, relapse site, tumor site, surgical margin, tumor size, grade and smoking history had no significant influence on disease free survival. Of note, we did not observe any differing survival between oral subsites (tongue or floor of mouth). None of the subjects from our study population had any distant metastases or extra-capsular invasion at presentation; therefore, these variables were not examined. A total of 68 out of 86 patients were treated by surgery only; the remaining received radiotherapy in addition to surgery. Disease free survival did not differ significantly between the treatment groups (data not shown).

We also performed a multivariate Cox-regression analysis of disease free survival related to miR-21 expression levels in combination with other clinical parameters derived from the univariate analysis, which were shown to be of importance for DFS (Table 3) and performed backward elimination. In this adjusted model, perineural invasion, N-stage and miR-21 TBS remained independent prognostic factors, although perineural invasion was only borderline significant (P = 0.078).

**Table 3:** Multivariate model of factors associated with disease free survival.

	Adjusted hazard ratio (95% CI)	p-value
**MiR-21 TBS**	1.6 (1.1–2.5)	0.044*
**Perineural invasion**	2.1 (0.92–4.5)	0.078*
**N-status**	2.5 (1.2–5.6)	0.015

Legend: The remaining factors in multivariate Cox regression after backwards elimination. Only miR-21 TBS and perineural invasion remained as independent factors influencing survival.

## Discussion

In the present study, we correlated miR-21 expression specifically in the tumor stroma, measured as the product of miR-21 intensity and the area occupied by miR-21 (blue signal), TBS, with patient survival and found that patients with high miR-21 expression have a shorter disease-free survival than patients who have low miR-21 expression. Clinically we observed a correlation between gender and miR-21 expression which have previously been observed in colon cancer [27].

The distribution of miR-21 expression varies with tumor type [2]–[6], [11]–[13]. We found that in OSCC, miR-21 expression was primarily found in tumor stromal fibroblast like cells and in intra tumor vessels. A similar distribution has been found in colon and breast carcinomas [5], [15], [16]. However, esophageal carcinomas, which originate from stratified epithelia and resemble nonkeratinized oral mucosa, express miR-21 principally in the tumor cells [12], [13]. The biological basis for these differences is currently poorly understood.

The TBS measurement used to quantitate miR-21 expression (DB/R) discriminates between stroma and tumor. To exclude the possibility that the poor prognosis seen in the patients overexpressing miR-21 was simply related to a larger stromal component of their tumors, we also investigated whether increasing stromal tumor content was related to a reduced disease free survival. This was however seen not to be related to a decreased disease free survival. In addition we were able to quantitate the amount of miR-21 specifically in the epithelial tumor cell compartment, to see if this was related to disease free survival. However, miR-21 expression in the tumor compartment was not related to a decreased disease free survival. Thus, it is interesting that the poor prognosis related to miR-21 expression reflects a pathological process in the stromal compartment. This observation is in agreement with previous findings, which show that high levels of myofibroblasts in the stroma of OSCCs are strongly associated with increased mortality from oral carcinomas regardless of disease stage [18], [19]. The expression of miR-21 in myofibroblasts suggests that miR-21 positive cells are largely activated tumor-associated fibroblasts. Tumor-associated fibroblasts are partly activated by TGF-beta signaling, which both promotes the expression of miR-21 and the differentiation of myofibroblasts [28]. Thus, miR-21 expression in myofibroblasts is indicative of a parallel pathway of activation.

Myofibroblasts can be classified into two types. The first type is a protomyofibroblast, which is a partly differentiated fibroblast that contains actin stress fibers but no immunohistochemically detectable α-smooth muscle actin [29]. The second type expresses α-smooth muscle actin and is considered to be a mature myofibroblast. miR-21 is found in cell types other than myofibroblasts, which suggests that miR-21 is expressed in mature fibroblasts and precursor cells. This supposition is supported in recent studies, which demonstrated the importance of miR-21 in the development of myofibroblasts. Specifically, the progression from protomyofibroblasts to myofibroblasts could be blocked using anti-sense inhibitors of miR-21 [28]. However, miR-21 overexpression *alone* was not sufficient to drive the differentiation toward myofibroblasts [16] suggesting that miR-21 is necessary but insufficient for the differentiation of myofibroblasts.

The present study builds on prior studies, which have demonstrated that neoplastic progression is not solely determined by the cancer cells but also by stromal processes surrounding the tumor [29]. This fact suggests that the evaluation of the malignant tumor stroma has an important prognostic value. Thus, even when the tumor is removed within a “safe margin”, there may be left-over tumor-associated stromal tissue, which could facilitate a relapse of the malignant disease in a genetically altered but histologically normal adjacent oral mucosa [30].

Our data shows that miR-21 expression was in the center of the tumor and not at the invasive front. This finding is in accordance with previous studies on the distribution of myofibroblasts and fibroblast activating protein (FAP), another marker for activated fibroblasts, which is highly expressed within colon carcinomas and correlates with patient survival [31].

Histological investigation of tumors has shown that tumor budding, which are small clusters of tumor cells at the invasive front, is strongly associated with a poor patient survival [32]. Nonetheless, these cells are often not surrounded by tumor associated fibroblasts expressing miR-21 or α-smooth muscle actin [23]. Tumor budding is often closely correlated with lymph node metastasis, which was not found for miR-21 in this study, suggesting the possibility of two different disease mechanisms.

In addition to its function as a biomarker for disease free survival in OSCC, miR-21 could potentially be exploited as a therapeutic target. Promising in vivo studies have shown that anti-miR-21 PNAs could potently reduce miR-21 expression in xenograft models of breast cancer, which resulted in a reduced cancer growth [33].

In conclusion, to our knowledge we have for the first time provided the location of miR-21 and related this location to the presence of myofibroblasts in OSCC. In addition, we have shown that the expression of miR-21 in OSCC does not vary significantly with tumor site (tongue cancer or floor of the mouth cancer). We also find that increased miR-21 expression in the tumor stroma may be used as an independent prognostic biomarker (hazard ratio 2.7) for disease free survival in OSCC.
